# Enzootic Transmission of Yellow Fever Virus in Peru

**DOI:** 10.3201/eid0908.030075

**Published:** 2003-08

**Authors:** Juliet Bryant, Heiman Wang, Cesar Cabezas, Gladys Ramirez, Douglas Watts, Kevin Russell, Alan Barrett

**Affiliations:** * University of Texas Medical Branch, Galveston, Texas, USA; † Instituto Nacional de Salud, Lima, Peru; ‡ Ministry of Health, Lima, Peru; § U.S. Naval Medical Research Center Detachment, Lima, Peru

**Keywords:** Arbovirology, Peru, phylogeny, yellow fever virus, virus evolution, virus envelope proteins, research

## Abstract

The prevailing paradigm of yellow fever virus (YFV) ecology in South America is that of wandering epizootics. The virus is believed to move from place to place in epizootic waves involving monkeys and mosquitoes, rather than persistently circulating within particular locales. After a large outbreak of YFV illness in Peru in 1995, we used phylogenetic analyses of virus isolates to reexamine the hypothesis of virus movement. We sequenced a 670-nucleotide fragment of the prM/E gene region of from 25 Peruvian YFV samples collected from 1977 to 1999, and delineated six clades representing the states (Departments) of Puno, Pasco, Junin, Ayacucho, San Martin/Huanuco, and Cusco. The concurrent appearance of at least four variants during the 1995 epidemic and the genetic stability of separate virus lineages over time, indicate that Peruvian YFV is locally maintained and circulates continuously in discrete foci of enzootic transmission.

Yellow fever (YF) is an important reemerging arboviral disease and a cause of severe illness and death in South America and Africa. In South America, transmission of yellow fever virus (YFV) is characterized by two types of cycles: an urban pattern of interhuman transmission vectored by *Aedes aegypti* and a sylvatic cycle involving monkeys and forest canopy mosquitoes of *Haemogogus* and *Sabethes* genera. Urban YF has not been reported in South America since 1954 (*1*). However, the reinfestation of many densely populated coastal cities with *Ae. aegypti* indicates that surveillance and monitoring of endemic or epidemic YF viral activity remain critically important public health objectives. Central questions surrounding the ecology of YF have been the underlying factors that explain the cyclic appearance and disappearance of virus activity from certain locales and the means by which the virus survives between epidemics. Despite extensive efforts to study the vector and host cycles of YFV within South America (*2*) and reported associations of climatic variables with epizootic activity (*3*), the parameters that influence the sylvan transmission cycle and the factors that trigger emergence of outbreaks have been poorly understood.

The most widely accepted hypothesis of YFV ecology in South America is that the virus is maintained by “wandering epizootics” of nonhuman primate species that move continuously throughout the Amazon region or along gallery forests of the river courses. Virtually all New World primate species are highly susceptible to YFV infection, and many neotropical species die of the infection. The acute viremic phase in monkeys is followed by solid immunity, and although persistent infection has been documented for some primate species in the laboratory, such infections are probably not accompanied by viremia levels sufficient to infect vectors (*4*,*5*). In Panama, Trinidad, and Brazil, finding dead monkeys (particularly *Alouatta* sp.) near forested regions has signaled the onset of epizootics. Many researchers have suggested that epizootics are cyclical events recurring at fairly regular intervals; the length of interepidemic intervals has been interpreted as the time required for reconstitution of susceptible monkey populations (*6*,*7*). Efforts to show evidence of alternative vector and host cycles (other than primates) have not been successful (*1*,*2*).

Vertical transmission of YFV in the mosquito vector may contribute to virus maintenance in nature. Field studies in eastern Senegal (*8*) provided indirect evidence for vertical transmission of YFV by *Aedes furcifer-taylori* through isolation of virus from male mosquitoes; similar attempts to demonstrate vertical transmission in field-caught *Haemagogus* mosquitoes from Trinidad were unsuccessful (*1*). Because experimental studies demonstrating vertical transmission of YFV by *Aedes aegypti* (*9*,*10*) and *Haemagogus* (*11*), have demonstrated very low rates of vertical transmission, researchers have suggested that a primary arthropod-vertebrate is still necessary for virus amplification in nature. Ecologic monitoring for YFV is extremely difficult and expensive and has rarely been implemented for the vast area of the Amazon River Basin. In particular, YFV has never been isolated from any mosquito or wild-caught vertebrates in the Peruvian Amazon; hence, isolates collected from this region consist exclusively of human isolates from sporadic cases of disease.

In the absence of ecologic data regarding YFV infection rates in vector and vertebrate host populations, we have adopted molecular genotyping of the existing collection of human YFV isolates as a method to gain insight into the possible geographic dispersal patterns of YFV variants. We investigated the genetic diversity of YFV isolates obtained from infected humans in Peru over 22 years. We interpret the phylogenetic data in light of the concept of virus traffic in wandering epizootics. Our data suggest that the YFVs circulating in Peru have differentiated into several subpopulations, and rather than circulating as one intermixing (wandering) population, the variants appear to persist within discrete geographic foci of the Andean/Amazonian region.

## Materials and Methods

### Virus Strains and Origins

**Figure 1:**
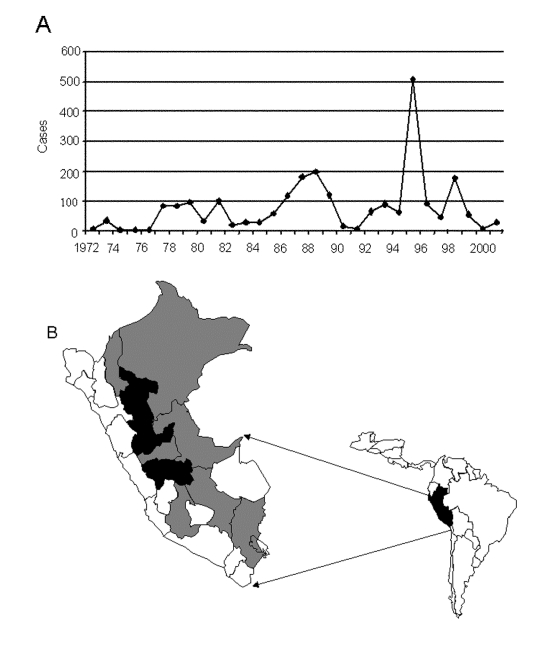
A) Peruvian river basins in which yellow fever virus is endemic. B) Annual incidence of confirmed cases of yellow fever in Peru, 1972–2001.

Twenty-five virus isolates were obtained from the World Arbovirus Reference Collection, at the University of Texas Medical Branch (UTMB), Galveston, Texas; the U.S. Naval Medical Research Center Detachment (NMRCD), Lima, Peru; and the Centers for Diseases Control and Prevention, Fort Collins, Colorado (Table 1). The viruses were originally isolated within the laboratories of the Instituto Nacional de Salud (INS) and NMRCD in Lima, at intervals from 1977 to 1999; with the exception of isolate #1914 from a sentinel mouse, the source material consisted of serum samples and tissue biopsy specimens from infected humans. To our knowledge, the 25 strains used in our study represent all strains of YFV isolated in Peru to date (*12*). The isolates represent 7 of the 14 hydrographic river basins identified as YF-endemic zones in Peru (Figure 1) (*13*). Thirteen of the 25 isolates (52%) were collected during the 1995 outbreak in Peru. Geographic coordinates for the YFV isolates were determined from case histories and reflected the communities in which the patients resided at the time of infection or the regional hospital at which they were treated. In cases for which neither the community nor the regional hospital was known, the largest population center of the locale was chosen as a reference. The ecozones and elevations associated with each of the locations of viral origin were obtained by using the original classification system of Holdridge (*14*) and the Mapa Ecologico de el Perú (*15*).

**Table 1:** Peruvian yellow fever isolates used in this study^a^

Strain ID	Date of illness onset	Sequence ID	Department	Community	Elevation^b^	Ecozone	Passage history^c^
1362/77	6/1977	PERU77A	Ayacucho	San Francisco	1,000–2,000	df-S or df-LM	c6/36#2
1368	6/1977	PERU77B	Ayacucho	Tribolina	1,000–2,000	vhf-S	SM1, Vero1, C6/36#2
1371	6/1977	PERU77C	Ayacucho	Chontacocha	0–1,000	hf-S	SM1, Vero1, C6/36#2
287/78	2/22/1978	PERU78	Ayacucho	San Francisco	1,000–2,000	sf-S	SM1, Mosq 2
R 35740	2/1979	PERU79	Ayacucho	Alto Montaro	0–1,000	vhf-S	SM1, Mosq 2
1899/81	6/19/1981	PERU81A	Cusco	Cusco	2,000–3,000	hf-M	SM1
1914^c^	6/12/1981	PERU81B	Cusco	Cusco	2,000–3,000	hf-M	LLCMK2, Vero 1, C6/36#1
ARVO544	1995	PERU95A	San Martin	Tocache Huaquisha	0–1,000	hf-T near vhf-S	SM1, Vero1, C6/36#2
HEB4224	1995	PERU95B	San Martin	Tocache Nuevo Progresso	2,000–3,000	hf-T near vhf-S, vhf-LM	SM1, C6/36#1
HEB4236	3/2/1995	PERU95C	Pasco	Oxapampa Villa Rica	1,000–2,000	hf-M	C6/36#1
149	3/95	PERU95D	Pasco	Oxapampa Villa Rica	1,000–2,000	hf-M	SM1, C6/36#1
Cepa#2	9/95	PERU95E	Puno	No data	2,000–3,000	hf-S	SM1, C6/36#1
Cepa#1	9/95	PERU95F	Puno	No data	2,000–3,000	hf-S	C6/36#2
OBS 2240	2/95	PERU95G	Huanuco	Hermil	1,000–2,000	vhf-LM	C6/36#2
OBS 2250	5/16/1995	PERU95H	Huanuco	Hermil	1,000–2,000	vhf-LM	SM1, C6/36#1
HEB 4240	1/30/1995	PERU95I	Junin	Chachamayo	1,000–2,000	hf-LM	C6/36#1, SM1
HEB 4245	3/6/1995	PERU95J	Junin	Chachamayo	1,000–2,000	hf-LM	SM1, C6/36#1
HEB 4246	3/8/1995	PERU95K	Junin	Chachamayo	1,000–2,000	hf-LM	SM1, C6/36#1
OBS 2243	2/95	PERU95L	Huanuco	No data	1,000–2,000	vhf-LM	SM1, C6/36#1
ARV 0548	3/19/1995	PERU95M	San Martin	Tocache Huaquisha	0–1,000	hf-T near vhf-S	SM1, C6/36#1
OBS 6530	3/26/1998	PERU98A	Cusco	Echarate	1,000–2,000	df-S	SM1, C6/36#1
03-5350-98	3/13/1998	PERU98B	Cusco	Kanaiquinaba	2,000–3,000	sf-S	C6/36#2
OBS 6745	3/29/1998	PERU98C	Cusco	Minsa/C.S. Moronacocha	1,000–2,000	hf-M	C6/36#2
IQT 5591	1/19/1998	PERU98D	Loreto	Belen, Tihuensa	0–1,000	hf-T	C6/36#2
OBS 7904	5/5/1999	PERU99	San Martin	Tarapoto	2,000–3,000	hf-S and vhf-S	Vero1, C6/36#3

^a^SM, suckling mouse; df-S, dry forest-subtropical; df-LM, dry forest-lower montane; vhf-S, very humid forest-subtropical; hf-S, humid forest-subtropical; sf-S, shrub forest-subtropical; hf-M, humid forest-montane; hf-T, humid forest-tropical; vhf-LM, very humid forest-lower montane; hf-LM, humid forest-lower montane. ^b^Elevation**,** range of meters above sea level for the ecozone immediately surrounding the place of viral origin. ^c^Passage history of seed strain in collection. ^d^Strain 1914 was obtained from a sentinel mouse.

### Sequence Determination

After transfer of the low passage isolates to the World Arbovirus Reference Center, viruses were grown for a single passage in Vero cells to obtain sufficient quantities for RNA extraction. Methods for viral growth, genomic RNA extraction, and amplification of viral sequences by reverse transcription polymerase chain reaction (RT-PCR) have been previously described (*16*). The genomic region under analysis comprised 670 nt of the premembrane (prM) and envelope (E) glycoprotein genes, using the genomic-sense primer (5′-CTGTCCCAATCTCAGTCC) and genomic-complementary primer (5′- AATGCTTCCTTTCCCAAAT). PCR products were screened by electrophoresis, recovered from gels using the QIAGEN gel extraction kit (QIAGEN, Valencia, CA), and sent for sequencing at the UTMB Protein Chemistry core facility. Sequences were obtained from both strands of each RT-PCR product for verification. The prM/E gene sequences obtained from the Peruvian YFV isolates have been deposited in GenBank (accession no. AY161927–AY161951).

### Phylogenetic and Statistical Analyses

Sequence editing and alignments were performed with Vector NTI (InforMax, Inc., Frederick, MD), and phylogenetic analysis was conducted by using PAUP* (*17*) and MRBAYES (*18*). Support for individual clades was determined by Bayesian inference with monte carlo markov chain simulation (*18*), as well as by nonparametric bootstrapping (*19*). To discern whether the pattern of genetic divergence was more closely related to geographic location or time of isolation, we generated matrices of pairwise comparisons of genetic, geographic, and temporal distances, and used Mantel’s test to evaluate correlations between the matrices. Geographic distances were calculated by using latitude and longitude coordinates and ArcView mapping software. The pairwise temporal-distance matrix was prepared by counting the months separating each pair of YF cases. Genetic distance matrices were generated by using the Kimura 2-parameter substitution model implemented in PAUP*. Mantel’s *Z* statistic and Pearson’s correlation coefficient (*r*) were calculated with MatMan version 1.0 (Noldus Information Technology, Wageningen, the Netherlands, 1998), and the significance of the *Z* statistic was computed by permutation analysis (10,000 repetitions).

To determine whether Peruvian YFVs were characterized by a single homogeneous rate of nucleotide substitution (i.e., a molecular clock), we performed a series of likelihood ratio tests using the PAUP* software analysis package. Maximum likelihood trees generated for the full dataset, as well as trees based exclusively on the third codon position, or on the first and second codon positions were also evaluated under the null model (no clock) and alternative models (molecular clock enforced).

## Results

### Sequence Variation among Peruvian YFVs

**Figure 2:**
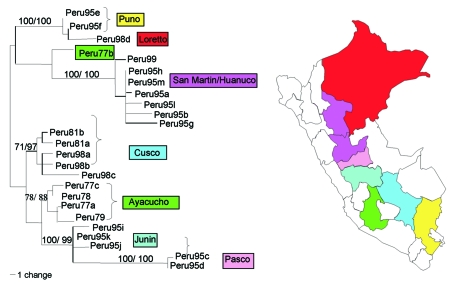
Maximum likelihood phylogeny of prM/E sequences of Peruvian yellow fever isolates constructed using PAUP*, 4.0b4a (*17*). Horizontal branch lengths represent genetic divergence, and numbers above the branch lengths denote support for individual clades as determined by nonparametric bootstrap analysis with 1,000 replicates (first value) and Bayesian posterior probabilities (second value). Only the values relevant for the interpretation of results are given. The strains used are listed in Table 1.

**Figure 3:**
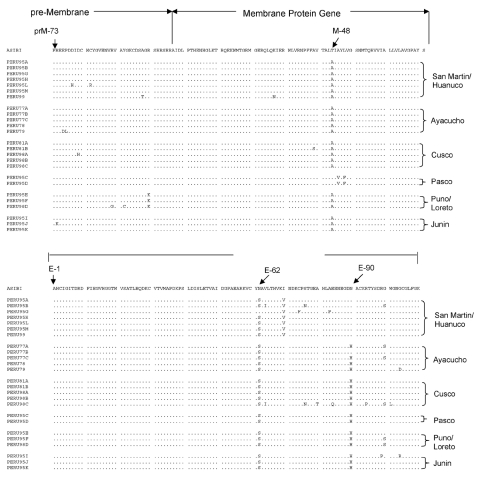
Amino acid alignment for the 25 Peruvian yellow fever virus. Dots indicate identity with prototype Asibi strain (from Ghana, 1927).

Figure 2 shows a maximum likelihood phylogeny based on the nucleotide sequences of the prM/E genes of 25 Peruvian YFV isolates. The Peruvian dataset contained 69 variable nucleotide positions, with a maximum of 7.3% nucleotide variability in all pairwise comparisons (average of 4.03%). Sixty of the nucleotide positions were parsimony informative; 48 informative sites occurred at third codon positions, whereas 12 informative sites occurred at first and second codon positions. Fifteen variable amino acids positions (6.7% of the 223 codons) were scattered throughout the prM, M, and E proteins (Figure 3). Pair-wise comparisons showed a mean of 1.87% amino acid variation (range 0% to 3.7%).

The phylogenetic tree of Peruvian YFV prM/E sequences showed six different clades that corresponded very closely with the geographic region of virus isolation and represented the states of Puno, Pasco, Junin, Cusco, Ayacucho, and San Martin/Huanuco (Figure 2). Three of the clades were distinguished by signature amino acid substitutions (i.e., coding changes in nucleotide sequences shared by all members of the group). The Puno strains shared a substitution in the premembrane protein (R→K prM102); the Pasco strains showed a triplet residue substitution motif in the membrane protein (A→T M48, A→V M50, and L→F M52); and the San Martin/Huanuco strains shared two residue substitutions within the envelope protein (I→V E72 and H→N E90) (Table 2). The remaining three clades (Ayacucho, Cusco, and Junin) were distinguished by silent nucleotide substitutions.

**Table 2:** Signature amino acid and nucleotide substitutions of Peruvian YFV genetic variants, based upon sequence analysis of prM-E gene fragments of 24 isolates

		Signature substitutions		
Department	No. of Isolates	nt	Amino acid	Coding changes from consensus of Peruvian strains
Ayacucho	5	1	0	None
Cusco	5	1	0	None
Junin	3	1	0	None
Puno	2	7	1	R→ K prM102
Pasco	2	3	3	A→T M48; A→V M50; L→F M52
San Martin/Huanuco	7	7	2	I→V E70; H→N E90

Because the sequence from one isolate did not group with those of its geographic neighbors (Ayacucho strain 1368, Peru 77b), the identity of this strain was subjected to additional scrutiny. Resequencing from original stock material, as well as consensus sequencing of populations that had been serially passaged three times in Vero cell culture, confirmed that the passaged strain was 100% identical to the parental population and that the sequence shared one of the signature amino acid substitutions characteristic of the San Martin/Huanuco clade. The single isolate from Loreto also showed an anomalous position on the tree; it appeared most closely related to the two Puno strains, which are paradoxically the most geographically distant. Reasons for the anomalous phylogenetic clustering of these isolates remain unclear.

### Correlation of Genetic Distance with Geography and Time

Following the approach taken by Bowen et al. (*20*), we used Mantel’s test to assess the strength of correlation between genetic variability, geographic distribution, and the times of virus isolation. We hypothesized that in a virus population circulating as a wandering epizootic, most of the genetic variation in isolates would be because of differences in the times of virus isolation. This finding is in contrast to the pattern of genetic variation expected from an enzootic transmission cycle; if subpopulations of viruses are isolated in discrete enzootic foci, one would expect genetic variation to correlate more closely to geographic rather than temporal distances. Our analysis of the variability among the 25 Peruvian YFVs showed a significant correlation between genetic and geographic distance (r=0.56, *Z*= 22898.51, p<0.0001), as well as between genetic and temporal distances (r=0.127, *Z*=147.04, p<0.05). The possibility of an interaction effect between geographic and temporal factors clearly exists, which was tested by performing a modified version of Mantel’s test. We calculated the partial rowwise Mantel *Zr* and partial Pearson’s *r* and found that after controlling for geographic distance, the correlation between the genetic and temporal matrices was no longer significant (r=0.057, Zr=0.073, p>0.1). In contrast, the correlation between genetic and geographic distances remained highly significant after controlling for temporal variation (r=0.55, Zr=0.55, p<0.0005). These results suggest that temporal factors alone do not adequately explain the divergence pattern of the Peruvian YFV phylogenetic tree. In contrast, the strong correlation between genetic and geographic distances indicates a high degree of population substructure and suggests that the clades of Peruvian YFVs may be evolving as separate distinct lineages.

### Molecular Evolution of YFV

Tests of “clocklike” behavior or uniformity in rates of evolution were introduced into our study as an additional method of assessing the amount of temporal structure in the sequence dataset. Under the molecular-clock assumption, the maximum likelihood branch lengths from the root of the tree to each branch tip are expected to be equal (i.e., the evolution changes in any lineage is expected to be proportional to elapsed time since divergence from a common ancestor). Results of the likelihood ratio tests for a molecular clock indicated that Peruvian YFVs in different subclades are evolving at different rates; trees constructed under the null model, allowing for rate heterogeneity, were assigned a statistically higher likelihood score than trees reconstructed with the molecular clock enforced. The lack of clocklike behavior in the phylogenetic reconstructions is a further indication of the lack of consistent temporal associations among Peruvian YFVs. Small populations are prone to genetic drift and “founders effects,” such as transmission bottlenecks; the combined effects of geographic isolation and small population size are the most likely explanations for the different branch lengths for each of the Peruvian clades. Therefore, the observation of different evolutionary rates (i.e., highly variable branch lengths) supports our hypothesis of limited gene flow and intermixing between of the YFV subpopulations in Peru.

## Discussion

Previous studies of the molecular epidemiology of YFVs have demonstrated that the virus genome exhibits remarkable stability, with low rates of genetic drift (*21*,*22*). The envelope gene region of YFV has been the focus of numerous molecular studies (*21*–*25*), and variability within the prM/E gene fragment appears to be representative of the virus genome (*24*,*25*). This study supports the observation that the coding sequence of YFV is highly conserved. The prM-E fragments, however, still retained sufficient phylogenetic signal to resolve several distinct geographic clades among the collection of Peruvian isolates. One unanticipated result of our study was the high degree of overall sequence divergence (7.3%) observed among the strains of the 1995 epidemic. The isolates collected during the 1995 epidemic varied at 71 nucleotide positions (10.7%) and represented four geographically distinct clades that could be distinguished by both silent and coding substitutions. This remarkable level of variability among strains from the same epidemic is unprecedented and approaches the threshold level of pairwise divergence (9%) previously used to distinguish the major genotypes of YFV in Africa (*25*). Molecular epidemiologic studies of related flaviviruses have previously observed very low levels of divergence among isolates from the same epidemic (*26*–*29*). Given the estimated substitution rate for YFV of ~5 x 10^-4^subs/site/y (based on the prM/E sequences of 38 African strains) (*25*), the numerous substitutions among the 1995 variants probably could not have accumulated during a single transmission season. Thus, our findings suggest that the large-scale epidemic in 1995 most likely resulted from concurrent emergence events in neighboring regions, rather than distribution and spread through epizootic waves of infection.

To our knowledge, our study has analyzed every available YFV isolate from Peru. The first isolates from Peru were obtained in 1977, and epidemic surveillance and outbreak investigations have yielded a total of only 25 isolates since that time. One of the difficulties of conducting molecular epidemiologic studies on the basis of historical samples is the lack of balanced, representative datasets. Given the severe limitations of the number of available YFV isolates from Peru, considerable caution must be exercised when inferring evolutionary implications from the observed genetic variability. Unfortunately, only three of the six Peruvian clades contain representatives from different time points. Strains collected from Ayacucho, San Martin/Huanuco, and Cusco represent intervals of 2, 4, and 17 years, respectively; isolates from these regions have diverged by <0.3% (Figure 2). The stability of these clades over a period of almost 2 decades suggests limited levels of exchange between virus subpopulations of adjacent watersheds. We propose that our ability to discern separate clades is possible because significant gene flow between the subpopulations has not occurred, as one might expect from virus traffic in wandering epizootics.

The original concept of wandering epizootics to explain YFV transmission cycles in South America grew out of observations of the cyclical occurrence and disappearance of outbreaks within particular geographic areas. The concept of continual virus movement has prevailed over the past years in part because of the failure to identify a vertebrate reservoir, and in part because of the dearth of ecologic data to support or refute alternative modes of transmission (*29*). Epizootics may appear to be wandering if disease incidence peaks in one area, sweeps through a population, then disappears for a period while the focus of transmission moves onward to a distant location. Epidemiologic data on YF incidence in Peru do not support a pattern of virus movement. Figure 1 presents incidence data for YF cases within different departments of Peru and demonstrates that sporadic cases occur on an annual or semiannual basis within tropical and subtropical montane forests (*30*). These observations indicate that no interval exists that can be construed as an interepidemic period during which the virus is completely absent; hence, transmission most likely occurs continuously within specific locales. While monkey epizootics are frequently reported in association with Brazilian outbreaks of YF (*31*), no instances of monkey deaths have been reported during Peruvian epidemics. Population subdivision among YFV from different river basins could be explained as an example of vicariant evolution, and as such, would lend further support to the hypothesis that other non-primate vertebrate hosts or vertical transmission by mosquito vectors, plays a role in maintaining an unbroken virus cycle.

Whether the finding of localized enzootic foci is a unique characteristic of YF transmission cycles within the montane regions of the Andes remains unclear. Patterns of focal endemicity of YFV have been suggested for certain areas of Brazil on the basis of repeated annual isolations of YFV from *Haemagogus janthinomys* during the rainy season along secondary roads of the TransAmazon Highway (*3*). However, the periodic appearance and disappearance of YF outbreaks observed in the eastern regions of Brazil and the genetic variation among Brazilian YFVs indicate that transmission cycles of that region are more broadly distributed than those of Peru (P.F.C. Vasconcelos, pers. comm.). Future molecular typing of YFV isolates from the widespread 2000 epizootic in Brazil will help clarify patterns of virus dissemination.

Climatic changes with increase in rainfall were previously associated with YF epidemic and epizootic episodes (*3*). Abundant evidence has accumulated regarding the critical role of rainfall and temperature in altering the vectorial capacity of mosquitoes, and such factors may have played a role in the 1995 outbreaks. Interestingly, however, neither excessive rainfall nor increased temperatures associated with El Nino and southern oscillation events were reported during 1995 in Peru (*32*). One potential factor that may have influenced the 1995 outbreaks was the increase in internal human migration that began in 1994 in association with the opening up of new agricultural and industrial areas in enzootic areas (*33*). During the early 1990s, the Peruvian government instituted economic reforms that led to rapid privatization of many government-run mining and agricultural operations. Economic dislocations resulted in acceleration of the ongoing massive movement of highland Indians into the cities of the coast and the settlements of the lowland forests to the east. Mass migrations and increased clearing of land may have changed the exposure status of human populations within endemic regions. Movement of agricultural workers and colonists from areas not endemic for YF into the Amazon Basin is a continuing challenge for public health authorities responsible for immunization programs. The cutting of trees and clearing of forests for agriculture represents a high risk for human infection, since *Haemogogus* species in the rain forest canopy are brought to ground level with subsequent increase in mosquito-human contact. We suggest that the intrusion of humans upon the usually silent sylvatic transmission cycle of the Peruvian Amazon was most likely the underlying reason for multiple concurrent outbreaks that occurred in 1995.

**Figure 4:**
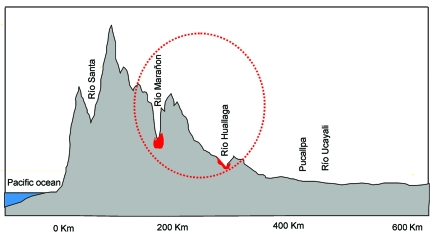
Schematic topographic cross-section through the southern Peruvian Amazon. Areas in red indicate river basins where yellow fever virus is endemic.

The genetic diversity of Peruvian YFV is best interpreted in light of the remarkable biologic diversity and unique ecologic features of Andean premontane and montane rain forest. Peru is in many respects one of the most diverse countries of the world; the geographic territory of Peru contains 84 of the 103 ecologic life zones proposed by Holdridge for the world (*15*), and the terrain of the eastern Andean foothills is renowned for its extraordinary topographic complexity and numerous centers where endemic species are found. Estimates published in Instituto Nacional de Salud (*30*) and Pan American Health Organization (*33*) documents mention elevational limits of the YF endemic zone from 650 m to 1,000 m or 400 m to 200 m, respectively. Our estimates of the geographic origin of YFV isolates in our collection, however, suggest that YF activity may extend to elevations >2,000 m. A considerable portion of the YF endemic zone would therefore be expected to comprise either premontane forest (from 500 m to 1,500 m) or humid montane forest (1,500 m to 3,500 m), as these are the vegetation formations in the states of Amazonas, San Martin, Huanuco, Pasco, Junin, Ayacucho, Cusco, and Puno, and the adjacent portions of Apurimac and Madre de Dio (*34*). We speculate that the population structure of Peruvian YFV may have resulted from geographic isolation of vertebrate host or mosquito vectors. The complex topography of these regions limits the range and dispersal of many organisms, and may likewise present strong barriers to virus dispersal (Figure 4). As more data become available from biologic surveys of YF-endemic areas, insight into the possible vertebrate reservoirs and arthropod vectors responsible for maintenance of the virus in nature may be gained.

Our study represents an attempt to apply the relatively new techniques of molecular genotyping to address long-standing, intransigent questions regarding YFV transmission cycles in nature. Despite substantial uncertainty regarding the ecologic parameters of the transmission cycle, our results suggest that YFV has a highly restricted pattern of geographic distribution within Peru. The limited number of strains available from Peru indicate that the transmission cycle most likely involves a persistent enzoosis that is either vertically maintained within arthropod populations or that survives as a latent infection within an as yet unidentified vertebrate host. The full extent of the geographic distribution of YFV variants within Peru will become clearer in the future, as additional isolates become available for analysis. The finding that YF transmission remains relatively limited within discrete enzootic foci lends support to the argument for highly focused interventions in the event of future outbreaks.
